# Decomposing the causes of socioeconomic-related health inequality among urban and rural populations in China: a new decomposition approach

**DOI:** 10.1186/s12939-017-0624-9

**Published:** 2017-07-18

**Authors:** Jiaoli Cai, Peter C. Coyte, Hongzhong Zhao

**Affiliations:** 1 School of Economics, Wuhan University of Technology, 122 Luoshi Road, Wuhan, 430070 Hubei Province People’s Republic of China; 2 Institute of Health Policy, Management and Evaluation, University of Toronto, Health Sciences Building, 155 College Street, Suite 425, Toronto, ON M5T 3M6 Canada

**Keywords:** Health inequality, Decomposition analysis, Concentration index, Socioeconomic status

## Abstract

**Background:**

In recent decades, China has experienced tremendous economic growth and also witnessed growing socioeconomic-related health inequality. The study aims to explore the potential causes of socioeconomic-related health inequality in urban and rural areas of China over the past two decades.

**Methods:**

This study used six waves of the China Health and Nutrition Survey (CHNS) from 1991 to 2006. The recentered influence function (RIF) regression decomposition method was employed to decompose socioeconomic-related health inequality in China. Health status was derived from self-rated health (SRH) scores. The analyses were conducted on urban and rural samples separately.

**Results:**

We found that the average level of health status declined from 1989 to 2006 for both urban and rural populations. Average health scores were greater for the rural population compared with those for the urban population. We also found that there exists pro-rich health inequality in China. While income and secondary education were the main factors to reduce health inequality, older people, unhealthy lifestyles and a poor home environment increased inequality. Health insurance had the opposite effects on health inequality for urban and rural populations, resulting in lower inequality for urban populations and higher inequality for their rural counterparts.

**Conclusion:**

These findings suggest that an effective way to reduce socioeconomic-related health inequality is not only to increase income and improve access to health care services, but also to focus on improvements in the lifestyles and the home environment. Specifically, for rural populations, it is particularly important to improve the design of health insurance and implement a more comprehensive insurance package that can effectively target the rural poor. Moreover, it is necessary to comprehensively promote the flush toilets and tap water in rural areas. For urban populations, in addition to promoting universal secondary education, healthy lifestyles should be promoted, including measures such as alcohol control.

**Electronic supplementary material:**

The online version of this article (doi:10.1186/s12939-017-0624-9) contains supplementary material, which is available to authorized users.

## Background

Understanding the temporal trends in health inequalities as well as their determinants are important for informed policy decision making that may reduce such health inequalities [1]. Health inequalities are differences in health that are potentially avoidable, unjust and/or unfair [2, 3]. They are mainly related to demographic and socioeconomic determinants, including age, gender, income and education [4–6]. Growing evidence indicates that lower socioeconomic status is associated with poorer health [7], and that health inequalities favor high-income groups [8–10]. Socioeconomic-related health inequities may be influenced by socioeconomic factors directly, or may be explained by other socioeconomic-related factors such as lifestyle factors [11, 12]. Socioeconomic gradients in access to health care also lead to inequalities in health [13]. Access to health services is concentrated among those at the upper end of socioeconomic spectrum [14, 15].

China’s rapid transition from a planned economy to a more market led economy has resulted in dramatic economic growth [16]. However, this period of economic growth has not been associated with an equivalent improvement in health [17]. The growing disparity in health between urban and rural regions and between the rich and the poor has caused dissatisfaction with socioeconomic-related health inequality [18]. The Chinese government has adopted a number of measures to address health inequalities. For instance, the launch of the New Rural Cooperative Medical Scheme (NRCMS) in 2003 was designed to reduce inequity in access to health care for the rural population. Although these reforms have offered some relief to the (rural) poor, concerns remain [19–21].

In order to achieve socioeconomic-related health equality, it is vital to determine the causes of the socioeconomic-related health inequality. Decomposing socioeconomic-related health inequality can help to uncover specific factors that are potentially modifiable by policy decision makers. The dominant decomposition approach, the Wagstaff decomposition method, was proposed by Wagstaff et al. [22] and has been used extensively in previous studies [9, 23]. However, there are potential concerns with this decomposition method. First, this decomposition method only explains the degree of variation in health rather than the covariance between health and socioeconomic rank [24, 25]. For example, a strict assumption of this method is rank ignorability [24]; Second, this decomposition method is only applicable to absolute inequality indices, such as the absolute concentration index (even though it was developed for the relative concentration index) [24, 26]; Third, this decomposition method imposes many restrictive assumptions, such as rank ignorability and weighting function ignorability, which in empirical practice often are unreasonable to impose [26]; Forth, it is unclear how to actually interpret the parameters, and the so called contributions, within these decompositions [24, 26]. In this paper, therefore, we used a new decomposition approach, called recentered influence function (RIF) regression decomposition method, as proposed by Heckley et al. [26] to decompose socioeconomic-related health inequality in China. RIF regression decomposition has a few important benefits. First, this method explains the causes of socioeconomic-related health inequality by directly decomposing the weighted covariance of health and socioeconomic rank [26]; Second, this method is able to decompose all forms of inequality measures, such as the Erreygers index (EI) [24], the Wagstaff index (WI) [22], the standard concentration index (CI), the absolute concentration index (AC), the attainment-relative concentration index (ARCI), and the shortfall-relative concentration index (SRCI) [26, 27]; Third, this method requires fewer, and less restrictive assumptions than the Wagstaff decomposition method. For example, this method simultaneously relaxes the rank and weighting function ignorability assumptions [26]; Fourth, this new decomposition approach is simple to estimate and the results are easy to interpret [26, 28]. To our knowledge, this is the first study to use this new method to decompose socioeconomic-related health inequality in China. Given the dual structure of urban and rural areas in China, it is necessary to distinguish the different potential causes of socioeconomic-related health inequality in urban and rural populations. Therefore, the objectives of this study were to examine changes in socioeconomic-related health inequality in China from 1991 to 2006; and to explore the potential causes of socioeconomic-related health inequality in urban and rural areas. These findings may provide a reference for the Chinese government to reduce socioeconomic-related health inequality and promote health in China.

Our research may make at least two contributions to the existing literature. First, we used a new decomposition approach to examine socioeconomic-related health inequality in China and compared our findings to previous studies that used the Wagstaff decomposition method. Second, using data from the China Health and Nutrition Survey (CHNS), one of the longest running panel studies, we assessed the changes in health inequality among urban and rural populations for the period 1991 to 2006, the period over which health status data were available.

## Methods

### Data

The data used in this paper were derived from the CHNS survey, which was collected by the Carolina Population Center (CPC) at the University of North Carolina at Chapel Hill, the Institute of Nutrition and Food Hygiene, and the Chinese Academy of Preventive Medicine. The survey covered nine provinces (Liaoning, Heilongjiang, Shandong, Jiangsu, Henan, Hubei, Hunan, Guangxi and Guizhou) out of 31 provinces in China, thereby accounting for about 42% of China’s total population [9]. A multi-stage stratified sampling method was employed in the survey to choose the sample. The CHNS is a longitudinal survey conducted over nine waves in 1989, 1991, 1993, 1997, 2000, 2004, 2006, 2009 and 2011. But the data related to health status were not recorded in 1989, 2009 and 2011. Therefore, the current study was based on the remaining six waves, from 1991 to 2006. 63% of the respondents who participated in 1989 remained in the last round of the survey conducted in 2006 [29]. The response rate was high (averaging 88%) for each survey wave [9]. Knowing that the health characteristics and the influencing factors for health were different among children and adults, our sample was limited to individuals who were at least 18 years of age. There were some missing data in the sample. But based on the Little’s chi-squared test [30] and a series of independent t-tests [31], we found that the data that were missing were missing at random. So there was no difference between the missing cases and the complete cases. Starting with an overall sample of 52,114 adult respondents, after excluding the missing data, 47,939 respondents were included in the final sample, thereby representing 92.0% of the overall sample. There were 15,981 urban respondents and 31,958 rural respondents.

### Measuring socioeconomic-related health inequality

There are a range of measures of health inequality and a range of factors that may influence health inequality. As was shown in the introduction, socioeconomic-related health inequality was very prominent over the study period. Thus, this study aimed at decomposing the causes of socioeconomic-related health inequality. One particular measure concerns the concentration index, which is widely used for measuring socioeconomic-related health inequality [32–34]. The standard concentration index (CI), denoted below by CI, can be written as

1 $$ CI=\frac{2}{ n\mu}\sum_{i=1}^n{h}_i{R}_i-1 $$

where *h*_*i*_ is the good health indicator for individual *i* with the mean denoted as *μ* . *R*_*i*_ is the relative rank of the *i*th individual in the income distribution. *N* is the sample size. The value of CI varies between −1 and 1. A positive CI indicates that good health is more concentrated among high-income groups, i.e. pro-rich health inequality.

### RIF regression decomposition

As we mentioned in the introduction, there are potential concerns with the Wagstaff decomposition method. So Heckley et al. [26] proposed a new method - RIF regression method to decompose socioeconomic-related health inequality. The RIF is derived from the influence function (IF) [26]. Firpo et al. [28] developed the concept of the RIF and RIF regression. Heckley et al. [26] further proposed RIF regression decomposition to decompose socioeconomic-related health inequality (concentration index). Decomposition of the concentration index is performed by a two-step procedure: first, calculation of the RIF of the concentration index; and second, regressing the RIF on a set of covariates yielding the marginal effects of the covariates on the index [26]. Assuming a linear relationship between the dependent variable and the independent variables means that the RIF is the dependent variable in an ordinary least square (OLS) regression whose coefficients equal the marginal effects of the covariates *X* on the concentration index [26]. This is referred to as RIF-CI-OLS decomposition. Therefore, following Heckley et al. [26], we used RIF-CI-OLS which is both simple and attractive from an operational perspective to conduct decomposition. Practical implementation of RIF-CI-OLS regression decomposition is straight forward by using software, such as Stata. The mathematical process of the decomposition by Heckley et al. [26] was presented in the Additional file 1. Readers can also refer to the study by Heckley et al. [26].

### Variables

#### Outcome measure: Health

Self-rated health (SRH), which had four categorical outcomes (poor, fair, good, and excellent SRH), was recorded in the survey. We transformed the categorical SRH measure into a continuous measure on the scale from 0 to 1 using the method proposed by Van Doorslaer and Jones [35]. There were three steps in completing this transformation. First, an ordered probit model was employed to regress SRH on a set of covariates (including demographic and socioeconomic-related variables, see Table 1). Second, predictions of the linear index were used to predict good health scores. Third, the predicted health scores from the ordered probit model can be re-scaled to the [0, 1] interval by using the equation:

2 $$ {H}_i=\frac{h_i^{\ast }- \min \left({h}_i^{\ast}\right)}{ \max \left({h}_i^{\ast}\right)- \min \left({h}_i^{\ast}\right)}\kern0.5em $$

where ${h}_i^{\ast }$ is the predicted and continuous health scores,$\max \left({h}_i^{\ast}\right)$ is the maximum predicted health score, while $\min \left({h}_i^{\ast}\right)$ is the minimum predicted health score. The resulting *H*_*i*_ represents the new health score which has been be re-scaled to the [0, 1] interval. The larger is *H*_*i*_, the healthier the respondent.

**Table 1:** Descriptive statistics of the sample

Variable	Category	Urban (*n* = 15,981)		Rural (*n* = 31,958)		*P*-value
		Mean	SD	Mean	SD	
New health score	Higher score indicates better health.	0.525	0.163	0.550	0.171	<0.0001
SRH	1 = Poor; 2 = Fair; 3 = Good; 4 = Excellent	2.702	0.741	2.772	0.752	<0.0001
*Demographic characteristics*						
Male	Male = 1; Female = 0	0.476	0.499	0.479	0.500	0.0352
Age group	Quartile 1 (18–33)	0.242	0.428	0.258	0.438	0.0001
	Quartile 2 (33–43)	0.223	0.416	0.240	0.427	<0.0001
	Quartile 3 (43–56)	0.257	0.437	0.271	0.444	0.0015
	Quartile 4 (56+)^a^	0.279	0.448	0.232	0.422	<0.0001
Ethnicity	Han = 1; Ethnic Minority = 0	0.914	0.281	0.849	0.358	<0.0001
Marital status	Married	0.800	0.400	0.817	0.387	<0.0001
	Single	0.125	0.331	0.114	0.317	0.0002
	Divorced, separated, or widowed^a^	0.075	0.263	0.069	0.254	0.0363
Household size		3.738	1.423	4.166	1.591	<0.0001
*Health insurance*	Yes = 1; No = 0	0.491	0.500	0.229	0.420	<0.0001
*Socioeconomic status*						
Per capita household income	Log form	8.546	0.953	8.017	0.962	<0.0001
Work status	Work = 1; Not working = 0	0.620	0.486	0.774	0.419	<0.0001
Education degree	Primary^a^	0.384	0.486	0.543	0.498	<0.0001
	Secondary	0.538	0.499	0.443	0.497	<0.0001
	Tertiary	0.078	0.268	0.013	0.114	<0.0001
*Lifestyle factors*						
Smoking history	Yes = 1; No = 0	0.321	0.467	0.332	0.471	0.0109
Drinking alcohol	Yes = 1; No = 0	0.384	0.486	0.335	0.472	<0.0001
BMI	Underweight^a^	0.069	0.253	0.080	0.271	<0.0001
	Normal weight	0.477	0.499	0.551	0.497	<0.0001
	Overweight	0.327	0.469	0.275	0.447	<0.0001
	Obese	0.112	0.315	0.082	0.274	<0.0001
Tap water	Yes = 1; No = 0	0.918	0.274	0.577	0.494	<0.0001
Flush toilet	Yes = 1; No = 0	0.620	0.485	0.212	0.409	<0.0001
Region	Eastern^a^	0.410	0.492	0.404	0.491	0.0911
	Middle	0.337	0.473	0.331	0.470	0.1648
	Western	0.253	0.435	0.266	0.442	0.0028
Year	1991^a^	0.156	0.363	0.171	0.376	<0.0001
	1993	0.149	0.356	0.162	0.369	0.0001
	1997	0.164	0.370	0.153	0.360	0.0040
	2000	0.163	0.369	0.163	0.369	0.9189
	2004	0.189	0.391	0.175	0.380	0.0001
	2006	0.180	0.384	0.176	0.380	0.2191

^a^represents the reference group

*SRH* Self-rated health, *BMI* Body mass index

#### Explanatory variables

The literature has identified several sets of explanatory variables used to explain variations in health. There were demographic characteristics [7], socioeconomic status [36], lifestyle factors [11] and home environment [37]. Gender, age, ethnicity, marital status, place of residence and household size were included in demographic characteristics. Socioeconomic status factors included per capita household income, educational attainment and work status. Household income was expressed in 2006 Yuan prices (USD 1.00 = CNY 7.81) using the consumer price index to convert income into 2006 Yuan for consistency across years. Income was log transformed in order to reflect the non-linear relationship between health and income, as suggested by previous work [38]. Education was divided into three categories: primary education (including primary or no education), secondary education (including middle or high school), and tertiary education (including college or higher education). Work status was divided into two classes: work and not working. Lifestyle factors included smoking history (yes or no); alcohol consumption (yes or no); and body mass index (BMI). BMI, which measures relative weight, was calculated from self-rated height and weight as weight divided by height squared (kg/m^2^). Based on guidelines for Asian populations’ BMI, BMI was classified as underweight (<18.5 kg/m^2^), normal weight (18.5–23 kg/m^2^), overweight (23–27.5 kg/m^2^), and obese (> = 27.5 kg/m^2^) [39]. Home environment included the sources of drinking water (tap water or not); and whether there exists flush toilet or not in house. In addition, health insurance was also included. Regions and years were also controlled. The nine provinces were divided into three groups based on the region’s level of economic development, namely Eastern China (including Liaoning, Heilongjiang, Shandong and Jiangsu), Middle China (including Henan, Hubei, and Hunan) and Western China (including Guangxi and Guizhou).

### Statistical analysis

First, we analyzed the distribution of good health across different income groups using the new health scores and original SRH (for the definition of SRH, see Table 1). T-tests were performed to examine whether statistically significant differences existed in the mean value of each variable between the urban and rural samples. Second, we calculated the CI from 1991 to 2006 to analyze the changes of socioeconomic-related health inequality in those years. Third, we decomposed socioeconomic-related health inequality by using RIF-CI-OLS decomposition approach. Last, in order to make the results more convincing, we decomposed socioeconomic-related health inequality based on other alternative indices of inequality measures. All analyses were performed using Stata, version 13.0.

## Results

### Descriptive analysis by urban and rural populations

Table 1 shows the descriptive statistics for the urban and rural populations in the total sample. The results of t-tests show that there were significant differences in the mean value of most variables between urban and rural sample. Rural population had a higher health score than urban population. The proportion of Han nationality was higher in the urban sample than that in rural sample. Household size among the rural sample was bigger than that for the urban sample. Moreover, urban respondents were more likely to have health insurance, report higher income and receive higher levels of education compared with their rural counterparts. The proportion of respondents who were currently working (including farm and non-farm work) was higher in rural areas compared with that in urban areas. Rural respondents were more likely to report being underweight than urban respondents; while urban respondents were more likely to report being overweight or obese. The urban sample had a higher proportion of using tap water and having a flush toilet than their rural counterparts.

### Socioeconomic-related health inequalities from 1991 to 2006

Table 2 shows the distribution of good health across different income groups. Whether using original SRH or new health scores, for both urban and rural populations, health status of the respondents in the higher-income groups was better than those in the lower-income groups. From 1991 to 2006, average health scores fell, except for the health score in 1993 and 1997 for urban and rural populations, respectively. Average health scores were greater for the rural population compared with that for the urban population. Table 3 presents the CI from 1991 to 2006. The positive CI indicates that good health was focused on high income groups, i.e. pro-rich health inequality. From 1991 to 2006, overall health inequality increased. The CI in urban and rural areas performed differentially. From a longer time period, the CI in urban areas increased from 0.0304 in 1991 to 0.0439 in 1997, but decreased from 0.0439 in 1997 to 0.0433 in 2006; while the CI in rural areas increased continuously from 0.0292 in 1991 to 0.0332 in 1997, and then reached 0.0624 in 2006. For the rural areas, the CI in 2006 was twice that of 1991. This indicates that, over the study period, there was variability in the change in health inequality in urban areas; while health inequality in rural areas increased continuously. In 1993, 2000, 2004 and 2006, health inequalities in rural areas were greater than those in urban areas. In the other two years, 1991 and 1997, however, health inequalities in urban areas were greater than those in rural areas.

**Table 2:** The distribution of good health across different income groups

Urban	New health score	Year	1991	1993	1997	2000	2004	2006
		Low quartile	0.4935	0.5443	0.4685	0.4407	0.4047	0.3643
		Second quartile	0.5718	0.5964	0.5238	0.4859	0.4309	0.4171
		Third quartile	0.5902	0.6185	0.5583	0.5117	0.4669	0.4566
		High quartile	0.5969	0.6327	0.5896	0.5541	0.5048	0.4787
		Mean	0.5685	0.6027	0.5439	0.5179	0.4789	0.4585
		N	2493	2375	2614	2601	3019	2879
	Original SRH score	Year	1991	1993	1997	2000	2004	2006
		Low quartile	2.6096	2.7659	2.6650	2.4651	2.5587	2.3943
		Second quartile	2.7648	2.7860	2.7230	2.6123	2.5932	2.5017
		Third quartile	2.7674	2.8217	2.7398	2.6991	2.6534	2.5580
		High quartile	2.8292	2.8358	2.7730	2.7769	2.6611	2.6650
		Mean	2.7645	2.8101	2.7326	2.6924	2.6386	2.6058
		N	2493	2375	2614	2601	3019	2879
Rural	New health score	Year	1991	1993	1997	2000	2004	2006
		Low quartile	0.5645	0.5557	0.5566	0.4994	0.4334	0.4035
		Second quartile	0.5970	0.5890	0.5946	0.5520	0.4772	0.4442
		Third quartile	0.6287	0.6242	0.6285	0.5739	0.5055	0.4897
		High quartile	0.6430	0.6502	0.6478	0.5972	0.5591	0.5427
		Mean	0.5881	0.5840	0.5990	0.5540	0.5004	0.4844
		N	5461	5190	4903	5213	5579	5612
	Original SRH score	Year	1991	1993	1997	2000	2004	2006
		Low quartile	2.7831	2.7702	2.7652	2.7019	2.5844	2.5288
		Second quartile	2.8566	2.8401	2.8379	2.7693	2.6036	2.5816
		Third quartile	2.8938	2.9034	2.9204	2.8268	2.6828	2.6244
		High quartile	2.9777	2.9659	3.0239	2.8306	2.8143	2.7930
		Mean	2.8319	2.8276	2.8617	2.7811	2.6849	2.6605
		N	5461	5190	4903	5213	5579	5612

*SRH* Self-rated health

SRH is defined in Table 1

The number of people in each group is 25% of the total population

The higher the score is, the better the health is

**Table 3:** Standard concentration index for urban and rural samples

Year	1991	1993	1997	2000	2004	2006
Urban CI	0.0304 (2493)	0.0269 (2375)	0.0439 (2614)	0.0442 (2601)	0.0471 (3019)	0.0433 (2879)
Rural CI	0.0292 (5461)	0.0310 (5190)	0.0332 (4903)	0.0450 (5213)	0.0508 (5579)	0.0624 (5612)
Total CI	0.0208 (7954)	0.0211 (7565)	0.0313 (7517)	0.0419 (7814)	0.0431 (8598)	0.0491 (8491)

Observations in brackets; CI, Standard concentration index

### Decomposing health inequality

Table 4 shows the results of decomposing health inequalities based on RIF-CI-OLS decomposition, indicating the effect of different factors on total socioeconomic-related health inequalities. Demographic characteristics, socioeconomic status, health insurance, lifestyles and home environment played different roles in influencing socioeconomic-related health inequality in urban and rural areas. For the urban population, respondents aged 18–33 and those who were married or single were negatively associated with health inequality. An increase in income decreased health inequality for urban population. Having health insurance and receiving secondary education were negatively correlated with health inequality. Respondents, who were Han nationality, were employed, received tertiary education, drank alcohol and were underweight or obese positively impacted health inequality. Western region was negatively related to health inequality. Health inequality increased over the study period. Gender, household size, smoking history, access to tap water and having a flush toilet had no effect on health inequality.

**Table 4:** RIF-CI-OLS decomposition estimates of covariates on socioeconomic-related health inequality

Variables	Category	Urban		Rural	
		Coef.	*P*-value	Coef.	*P*-value
*Demographic characteristics*					
Gender	Female	Reference		Reference	
	Male	−0.001	0.783	−0.011***	0.000
Age group	Quartile 4 (56+)	Reference			
	Quartile 1 (18–33)	−0.031***	0.000	−0.049***	0.000
	Quartile 2 (33–43)	−0.008	0.113	−0.020***	0.000
	Quartile 3 (43–56)	0.005	0.273	−0.012***	0.000
Ethnicity	Ethnic Minority	Reference		Reference	
	Han	0.015***	0.005	−0.007**	0.020
Marital status	Divorced, separated,and widowed	Reference		Reference	
	Married	−0.055***	0.000	−0.039***	0.000
	Single	−0.023***	0.002	−0.036***	0.000
Household size		0.001	0.244	−0.001	0.108
*Health insurance*	Yes = 1; No = 0	−0.013***	0.000	0.012***	0.000
*Socioeconomic status*					
Per capita household income		−0.048***	0.000	−0.026***	0.000
Work status	Work = 1; Not working = 0	0.036***	0.000	0.018***	0.000
Education degree	Primary education	Reference		Reference	
	Secondary education	−0.048***	0.000	−0.009***	0.000
	Tertiary education	0.039***	0.000	0.102***	0.000
*Lifestyle factors*					
Smoking history	Yes = 1; No = 0	0.003	0.451	−0.001	0.657
Drinking alcohol	Yes = 1; No = 0	0.009***	0.007	0.005*	0.064
BMI	Normal weight	Reference		Reference	
	Underweight	0.050***	0.000	0.046***	0.000
	Overweight	−0.004	0.231	−0.007	0.258
	Obese	0.015***	0.001	0.000	0.988
*Home environment*					
Tap water	Yes = 1; No = 0	−0.002	0.680	−0.016***	0.000
Flush toilet	Yes = 1; No = 0	−0.015	0.302	−0.009***	0.001
Region	Eastern	Reference		Reference	
	Middle	−0.003	0.401	−0.004	0.124
	Western	−0.010**	0.016	−0.005*	0.065
Year	1991	Reference		Reference	
	1993	0.003	0.490	0.010***	0.002
	1997	0.045***	0.000	0.029***	0.000
	2000	0.062***	0.000	0.039***	0.000
	2004	0.061***	0.000	0.033***	0.000
	2006	0.045***	0.000	0.024***	0.000
Constant		0.420***	0.000	0.246***	0.000
Observations		15,981	15,981	31,958	31,958

*** *p* < 0.01, ** *p* < 0.05, * *p* < 0.1

*Coef* Coefficient, *BMI* Body mass index

For the rural population, male, younger respondents, those who were of Han nationality and who were married or single were negatively associated with health inequality. An increase in income reduced health inequality. Respondents who received secondary education negatively influenced the inequality index compared with those who received primary education. Access to tap water and having a flush toilet helped to decrease the inequality index. Health insurance, work, the degree of tertiary education, drinking alcohol and being underweight were positively associated with health inequality. The Western region was negatively associated with health inequality. Health inequality rose over years. Household size and smoking history had no effect on inequality. We also decomposed health inequality based on other indices of inequality. The results were shown in the Tables 5 and 6 and they were consistent with the results in Table 4.

**Table 5:** RIF-I-OLS decomposition of other forms of rank dependent inequality indices for urban samples

Variables	Category	(1)	(2)	(3)	(4)	(5)
		rifEI	rifWI	rifARCI	rifSRCI	rifAC
*Demographic characteristics*						
Gender	Female (Reference)					
	Male	−0.001	−0.001	−0.001	−0.000	−0.000
Age group	Quartile 4 (56+) (Reference)					
	Quartile 1 (18–33)	−0.049***	−0.048***	−0.031***	−0.017***	−0.012***
	Quartile 2 (33–43)	−0.005	−0.004	−0.008	0.004	−0.001
	Quartile 3 (43–56)	0.015*	0.016*	0.005	0.011**	0.004*
Ethnicity	Ethnic Minority (Reference)					
	Han	0.032***	0.032***	0.015***	0.017***	0.008***
Marital status	Divorced, separated, or widowed (Reference)					
	Married	−0.115***	−0.115***	−0.055***	−0.060***	−0.029***
	Single	−0.042***	−0.042***	−0.023***	−0.019**	−0.011***
Household size		0.003	0.003	0.001	0.002	0.001
*Health insurance*	Yes = 1; No = 0	−0.029***	−0.029***	−0.013***	−0.017***	−0.007***
*Socioeconomic status*						
Per capita household income		−0.099***	−0.099***	−0.048***	−0.051***	−0.025***
Work status	Work = 1; Not working = 0	0.078***	0.079***	0.036***	0.043***	0.020***
Education degree	Primary education (Reference)					
	Secondary education	−0.098***	−0.097***	−0.048***	−0.049***	−0.024***
	Tertiary education	0.088***	0.089***	0.039***	0.050***	0.022***
*Lifestyle factors*						
Smoking history	Yes = 1; No = 0	0.007	0.007	0.003	0.004	0.002
Drinking alcohol	Yes = 1; No = 0	0.022***	0.022***	0.009***	0.013***	0.005***
BMI	Normal weight (Reference)					
	Underweight	0.102***	0.101***	0.051***	0.051***	0.025***
	Overweight	−0.005	−0.004	−0.004	−0.000	−0.001
	Obese	0.029***	0.029***	0.015***	0.014***	0.007***
*Home environment*						
Tap water	Yes = 1; No = 0	−0.005	−0.005	−0.002	−0.003	−0.001
Flush toilet	Yes = 1; No = 0	−0.033	−0.033	−0.015	−0.018	−0.008
Region	Eastern (Reference)					
	Middle	−0.009	−0.010	−0.003	−0.007*	−0.002
	Western	−0.024***	−0.025***	−0.010**	−0.015***	−0.006***
Year	1991 (Reference)					
	1993	0.009	0.009	0.003	0.006	0.002
	1997	0.093***	0.093***	0.045***	0.048***	0.023***
	2000	0.127***	0.127***	0.062***	0.065***	0.032***
	2004	0.122***	0.122***	0.061***	0.061***	0.031***
	2006	0.088***	0.088***	0.045***	0.043***	0.022***
Constant		0.855***	0.854***	0.420***	0.434***	0.214***
Observations		15,981	15,981	15,981	15,981	15,981

*** *p* < 0.01, ** *p* < 0.05, * *p* < 0.1

*EI* Erreygers index, *WI* Wagstaff index, *ARCI* Attainment-relative concentration index, *SRCI* Shortfall-relative concentration index, *AC* Absolute concentration index, *BMI* Body mass index

**Table 6:** RIF-I-OLS decomposition of other forms of rank dependent inequality indices for rural samples

Variables	Category	(1)	(2)	(3)	(4)	(5)
		rifEI	rifWI	rifARCI	rifSRCI	rifAC
*Demographic characteristics*						
Gender	Female (Reference)					
	Male	−0.019***	−0.019***	−0.011***	−0.008**	−0.005***
Age group	Quartile 4 (56+) (Reference)	Reference				
	Quartile 1 (18–33)	−0.079***	−0.073***	−0.049***	−0.024***	−0.020***
	Quartile 2 (33–43)	−0.022***	−0.018**	−0.020***	0.002	−0.006***
	Quartile 3 (43–56)	−0.014**	−0.011	−0.012***	0.001	−0.003**
Ethnicity	Ethnic Minority (Reference)					
	Han	−0.016**	−0.016**	−0.007**	−0.010***	−0.004**
Marital status	Divorced, separated, or widowed (Reference)					
	Married	−0.083***	−0.084***	−0.039***	−0.045***	−0.021***
	Single	−0.075***	−0.075***	−0.036***	−0.039***	−0.019***
Household size		−0.002	−0.001	−0.001	−0.000	−0.000
*Health insurance*	Yes = 1; No = 0	0.024***	0.024***	0.012***	0.012***	0.006***
*Socioeconomic status*						
Per capita household income		−0.054***	−0.054***	−0.026***	−0.029***	−0.014***
Work status	Work = 1; Not working = 0	0.045***	0.047***	0.018***	0.028***	0.011***
Education degree	Primary education (Reference)					
	Secondary education	−0.025***	−0.027***	−0.009***	−0.018***	−0.006***
	Tertiary education	0.228***	0.231***	0.102***	0.129***	0.057***
*Lifestyle factors*						
Smoking history	Yes = 1; No = 0	−0.004	−0.004	−0.001	−0.003	−0.001
Drinking alcohol	Yes = 1; No = 0	0.014**	0.015**	0.005*	0.010***	0.003**
BMI	Normal weight (Reference)					
	Underweight	0.095***	0.095***	0.046***	0.049***	0.024***
	Overweight	−0.011	−0.01	−0.007	−0.003	−0.003
	Obese	0.002	0.003	0.000	0.003	0.001
*Home environment*						
Tap water	Yes = 1; No = 0	−0.035***	−0.036***	−0.016***	−0.020***	−0.009***
Flush toilet	Yes = 1; No = 0	−0.023***	−0.025***	−0.009***	−0.016***	−0.006***
Region	Eastern (Reference)					
	Middle	−0.017***	−0.020***	−0.004	−0.016***	−0.004***
	Western	−0.027***	−0.031***	−0.005*	−0.026***	−0.007***
Year	1991 (Reference)					
	1993	0.022***	0.022***	0.010***	0.012***	0.006***
	1997	0.064***	0.065***	0.029***	0.036***	0.016***
	2000	0.080***	0.080***	0.039***	0.041***	0.020***
	2004	0.065***	0.064***	0.033***	0.031***	0.016***
	2006	0.044***	0.043***	0.024***	0.020***	0.011***
Constant		0.505***	0.501***	0.246***	0.255***	0.126***
Observations		31,958	31,958	31,958	31,958	31,958

*** *p* < 0.01, ** *p* < 0.05, * *p* < 0.1

*EI* Erreygers index, *WI* Wagstaff index, *ARCI* Attainment-relative concentration index, *SRCI* Shortfall-relative concentration index, *AC* Absolute concentration index, *BMI* Body mass index

## Discussion and conclusion

China’s rapid economic development has led to major differences in social and economic life between urban and rural areas. Therefore, it is better to examine socioeconomic-related health inequality in urban and rural areas separately. Understanding the variation in health across different socioeconomic groups can help illuminate our understanding of the determinants of these inequalities. In the current study, we first compared the distribution of good health across different income groups. We then measured socioeconomic-related health inequality by calculating the conventionally employed CI between 1991 and 2006 in urban and rural areas. Finally, we analyzed the potential causes of these inequalities in urban and rural populations, separately. Several compelling new findings were revealed. First, our finding regarding the positive CI suggests that there was pro-rich health inequality in China. The rich had a greater share of good-health in China. Second, in the majority of the survey years, the CI in rural areas was slightly higher than that in urban areas, which suggests that health inequality in rural areas was slightly more pronounced than that in urban areas, and a higher degree of pro-rich health inequality existed in rural areas. Third, overall health inequality rose from 1991 to 2006. Fourth, through the decomposition of socioeconomic-related health inequality, we found that the potential causes of socioeconomic-related health inequality were different in urban and rural areas.

We found that the average level of health status declined from 1989 to 2006 for both urban and rural populations. This may be because urbanization triggers changes in occupational activities, social structures and socioeconomic status that may lead to illnesses such as cardiovascular disease, neuropsychiatric disorders, and other non-communicable chronic diseases [40]; or because environmental quality, including air and water pollution, contributes to lowering disease prevalence for both urban and rural areas [40, 41]; or because the transition to western style diets and sedentary lifestyles leads to a decline in health. One previous study reported that, today, China is faced with a set of health issues, such as the risks of hypertension, the impact of environmental pollution and smoking, and chronic and degenerative diseases [42]. Due to the absence of the relevant data, we were not able to examine the trend in various diseases or the trend in outdoor air pollution over the study period, however, we did find lifestyles and home environment had an impact on inequality in health.

Our finding that there existed pro-rich health inequality in China was consistent with previous studies on socioeconomic-related health inequality in China [43, 44]. This may be because that the rich can access more health resources. Zhang et al. [45] using the data from Chinese National Health Services Survey (NHSS) in 2008, found that there were substantial differences in health care utilization between the rich and the poor. Moreover, Zhou el al. [46] using the data from the Chinese NHSS in 1993, 1998, 2003 and 2008, found that with the same needs for health care, rich rural residents used more health care services than poor rural residents. In our study, we found that health inequality increased over the last two decades.

We also found that although in different years, there were small differences in the CI between rural and urban areas, the absolute value of the differences was less than 0.02. Consequently, these differences over the study period were small. In the majority of the survey years, the CI in rural areas was slightly higher than that in urban areas, which means that health inequality in rural areas was slightly more pronounced than that in urban areas. This finding was consistent with the findings by Xie [43]. Greater health inequality in rural China may be associated with the characteristics of rural areas. In China, health care systems were different for urban and rural populations. For the rural population, with the collapse of the Cooperative Medical System (CMS) in 1981, private health insurance was the only option for a long time [47, 48]. Private health insurance was expensive and unaffordable for most rural residents [49]. Financial barriers may widen health inequality for rural populations, especially for the rural poor. Despite the introduction of the New Rural Cooperative Medical Scheme (NRCMS) in 2003 for the rural population, it seems to have had a limited impact on improving access to formal care for the poor [50].

We also revealed some important causes of socioeconomic-related health inequality for urban and rural populations. Income was a common factor in reducing health inequalities in urban and rural areas in our study. In previous studies, income was often found to be associated with health and high-income groups tended to report good health [7, 12, 36, 51]. Zhou et al. [52] using data from the Chinese NHSS conducted in 2003 and 2008, reported strong pro-rich inequity in access to inpatient utilization in rural China. They found that income was the main determinant of this pro-rich inpatient utilization inequity, as wealthier people could pay for more services, and therefore, used more services regardless of need. Consequently, general improvements in income helped to reduce inequity in inpatient utilization. Another study also found that being poor prevented people from seeking care [53]. Thus, in order to promote health equality, it is important to provide support for the poor.

The level of education had a significant effect on health inequality for both urban and rural populations. However, secondary education helped to reduce inequality, while tertiary education promoted inequality in our study. Previous studies on the impact of education on health have yielded different findings. Cott et al. [54] found that those with lower level of education were less likely to report excellent or very good health. Prus [55] compared the determinants of SRH across the United States and Canada and found that the education gradient was steeper in the U.S compared with Canada. Yang and Kanavos [44] reported that education made an important contribution to total health inequality. However, because Yang and Kanavos used the Wagstaff decomposition method, it is unclear how to actually interpret the contribution. Moreover, this method only focuses on the impact of education on health rather than the impact of education on socioeconomic-related health inequality. Chen et al. [9] assessed income-related health inequality and health achievement in children in China and found middle school enrollments could promote health achievement, but primary school enrollments showed no influence.

In our study, respondents who were currently working were more likely to positively impact health inequality than those who were not working, which is consistent with the finding by Xie [43]. It is possible that people who were engaged in work were richer compared with those without a job, so people who had a job were healthier [43]. Thus, work status further strengthened socioeconomic-related health inequality. Previous studies also found that having a job increased the likelihood of reporting excellent/good health; and work status also made a positive contribution to total health inequalities [43, 44]. In our study, the impact of work status on health inequality was greater in urban areas compared with that in rural areas. As was shown in the result section, the rural respondents who were currently working included those who were engaged in farm work and non-farm work. This results in a higher proportion of respondents reporting that they were currently working (including farm and non-farm work) than those in urban areas. Because the majority of the rural respondents reporting that they were currently working, were actually engaged in farm work, which leads to a low socioeconomic status. Thus, the differences in socioeconomic status between those who were currently working and those who didn’t work may be small for rural population; while the differences may be great for urban population. This helps explain why health inequality in rural areas caused by work status was relatively smaller compared with that in urban areas (as was shown in Table 4, the coefficient on work status was smaller in rural areas).

It is worth noting that health insurance in rural and urban areas may also affect health inequalities. Health insurance helped to reduce health inequality for the urban population; while it increased health inequality for the rural population. It is expected that health insurance provides support for urban populations to seek care. The Urban Employee Basic Medical Insurance (UEBMI) scheme for urban workers and the Urban Residents Medical Insurance (URMI) scheme for children and non-working urban residents are the main forms of health insurance in urban areas. However, for the rural population, as we mentioned above, they were uninsured for a long period following the collapse of CMS in 1981, and rural populations only had recourse to private insurance until the introduction of the NRCMS in 2003 [47, 48]. The NRCMS, introduced between 2003 and 2008, aims at providing insurance to rural residents. NRCMS is a voluntary program that covers only those who join. However, evidence has shown that implementation of NRCMS has not improved the health of the rural population. Sun et al. [56] found that the NRCMS had little effect on reducing household health spending. Yang [50] found that the impacts of the NRCMS on improving access to formal care for the poor were limited. Similar findings were reported by Yu et al. [57], who found that under NRCMS inpatient service utilization has increased for high income groups, but there was no significant change for middle and low income groups, and people with higher incomes tended to benefit more than those on lower incomes. Several reasons may explain why NRCMS has had a limited impact on improving access to health services for the poor in rural areas. First, the low reimbursement rate and the high co-payments were an important limit to the success of the NRCMS. Under the NRCMS, the average level of national reimbursement for outpatient care was approximately 10% of overall expenses [58]. Similarly, the use of preventive care was unequally distributed and related to the unequal distribution of income level [50]. Second, low income participants were already burdened with a premium, while substantial co-payments due to the limited coverage further aggravated inequity in health care access [50]. The low reimbursement rate of the NRCMS and the associated financial burden from co-payments prevented the rural poor from seeking care [52, 59–61]. Thus, it is necessary to develop more comprehensive forms of coverage for outpatient and preventive care; and implement a more comprehensive insurance package to effectively target the rural poor and to provide low income participants with better financial protection. For services not being covered by the NRCMS, using commercial health insurance to improve equity may be an option for the rural populations [50]. Evidence of adverse selection may also help to explain why health insurance in rural areas did not contribute to reducing inequality; i.e., having sick household members increased the likelihood of enrollment in NRCMS [21, 62].

Finally, it should be noted that unhealthy lifestyles could increase health inequality for urban and rural populations. This may be because that people with different socioeconomic status have different lifestyles, thus influencing health [55]. Poor lifestyles were reported to result in ill health [63, 64]. The better home environment contributed to the reduction of health inequality in rural areas, but had no influence on the health inequality in urban areas.

This study had some potential limitations. First, there may exist other potential factors affecting health inequality, which we were unable to examine due to the limitations of the survey. For example, the distance to the medical institution may influence the willingness of people to seek medical care, thus resulting in disparities in health. However, we have incorporated as many potential factors as possible. Second, the data we used were drawn from nine Chinese provinces, representing 42% of total population of China [9]. However, the CHNS is one of the longest running panel studies, which provides an excellent opportunity to examine changes in health inequality for urban and rural Chinese residents over the past two decades. The nine provinces included in the survey are broadly representative of the economic and regional make-up of China. Third, SRH is a subjective measure, which may suffer from potential response bias. However, this limitation cannot be avoided in the absence of an objective measure of health.

In conclusion, our study provides a reference for the development of policies to reduce socioeconomic-related health inequalities. First, the key to addressing socioeconomic-related health inequalities is to increase the income of those with lower socioeconomic status, thereby improving their access to health services. Second, health insurance coverage could be expanded and vulnerable groups (such as older people and rural poor) could be included in the health care security net. Third, based on our findings, the overall health of the population declined over the past two decades and lifestyles and home environment played roles in this process; this finding suggests that more attention could also be paid to improvements in the lifestyles and home environment of residents while striving to improve income and enhancing access to the health care system. It is worth noting that an emphasis on empowering older people and improving healthy lifestyles from a very early age may be also result in cost-savings and reduce health inequality. Finally, some specific suggestions are stressed in order to reduce health inequality for the rural and urban populations. For the rural populations, the reform of health insurance and improvement of flush toilets and access to tap water require particular attention; while for urban populations, it is necessary to advocate for healthy lifestyles in order to prevent obesity and the occurrence of chronic disease.

## Additional file

Additional file 1: The mathematical process of RIF regression decomposition method. (DOCX 34 kb)

## Abbreviations

AC: Absolute concentration index
ARCI: Attainment-relative concentration index
BMI: Body Mass Index
CHNS: China Health and Nutrition Survey
CI: Standard concentration Index
CMS: Cooperative Medical System
CPC: Carolina Population Center
EI: Erreygers index
IF: Influence Function
NHSS: National Health Services Survey
NRCMS: New Rural Cooperative Medical Scheme
OLS: Ordinary Least Square
RIF: Recentered Influence Function
SRCI: Shortfall-relative concentration index
SRH: Self-rated health
UEBMI: Urban Employee Basic Medical Insurance
URMI: Urban Residents Medical Insurance
WI: Wagstaff index
