# Supplementary material for: Decomposing the causes of socioeconomic-related health inequality among urban and rural populations in China: a new decomposition approach
Source: Int J Equity Health. 2017 Jul 18;16:128. doi: 10.1186/s12939-017-0624-9 (PMC5516311; doi:10.1186/s12939-017-0624-9)
Supplement: Additional file 1: — The mathematical process of RIF regression decomposition method. (DOCX 34 kb) [file 12939_2017_624_MOESM1_ESM.docx]

Appendix 1

The mathematical process of RIF regression decomposition method proposed by Heckley et al.

Please note: The mathematical process below was derived from the paper of Heckley et al. [1]. Readers can also refer to the study by Heckley et al. [1].

***Measuring socioeconomic-related health inequality***

In order to represent different forms of health inequality measures, Heckley et al. (2016) [1] used a general form of the rank dependent inequality index to derive different forms of inequality indices.

The function of the general form of the inequality index ($I$) is as follows.

$I=v^{I}\left( F_{H,F_{Y}} \right) =v^{w_{I}}(F_{H}) v^{AC}(F_{H,F_{Y}})$ (1)

$H$ is the health indicator with probability measure denoted as $F_{H}$. $v^{w_{I}}(F_{H})$ is a weighting function specific to a particular form of inequality index. $Y$ is an individual’s income. The cumulative distribution function (CDF) of $Y$, $F_{Y}$, is the relative rank of the individual by income $Y$. The joint distribution of$H$ and $F_{Y}$ is denoted as $F_{H,F_{Y}}$. The absolute concentration index (AC) is given by the twice the covariance between $H$ and $F_{Y}$:

$AC= v^{AC}\left( F_{H,F_{Y}} \right)=2cov(H,F_{Y})$ (2)

When using standard concentration index (CI) in our study, then the weighting function is as follows:

$$v^{w_{CI}}\left( F_{H} \right)=\frac{1}{\mu_{H}} (3)$$

where $\mu_{H}$ is the mean of health variable $H$. Thus, the standard CI can be given as:

$$CI=v^{CI}\left( F_{H,F_{Y}} \right)=\frac{1}{\mu_{H}}2cov\left( H,F_{Y} \right) (4)$$

For Erreygers index (EI) [2], the weighting function is as follows:

$$v^{w_{EI}}\left( F_{H} \right)=\frac{4}{b_{H}-a_{H}} \left( 5 \right)$$

where the health variable of interest has both an upper and lower bound denoted as $b_{H}$ and as $a_{H}$ respectively.

Thus, the EI can be given as:

*EI=*$v^{EI}\left( F_{H,F_{Y}} \right)=\frac{4}{b_{H}-a_{H}}2cov\left( H,F_{Y} \right)$ (6)

For Wagstaff index (WI)[3], the weighting function is as follows:

$$v^{w_{WI}}\left( F_{H} \right)=\frac{b_{H}-a_{H}}{{(b}_{H}-\mu_{H})(\mu_{H}-a_{H})} (7)$$

Thus, the WI can be given as:

*WI=*$v^{WI}\left( F_{H,F_{Y}} \right)=\frac{b_{H}-a_{H}}{{(b}_{H}-\mu_{H})(\mu_{H}-a_{H})}2cov\left( H,F_{Y} \right) (8)$

For attainment-relative concentration index (ARCI) [1,4], the weighting function is as follows:

$$v^{w_{\mathrm{ARCI}}}\left( F_{H} \right)=\frac{1}{\mu_{H}-a_{H}} (9)$$

Thus, the ARCI can be given as:

*ARCI =*$v^{ARCI}\left( F_{H,F_{Y}} \right)=\frac{1}{\mu_{H}-a_{H}}2cov\left( H,F_{Y} \right) (10)$

For shortfall-relative concentration index (SRCI) [1,4], the weighting function is as follows:

$$v^{w_{\mathrm{SRCI}}}\left( F_{H} \right)=\frac{1}{b_{H}-\mu_{H}} (11)$$

Thus, the SRCI can be given as:

*SRCI =*$v^{SRCI}\left( F_{H,F_{Y}} \right)=\frac{1}{b_{H}-\mu_{H}}2cov\left( H,F_{Y} \right) (12)$

***The RIF for a general bivariate rank dependent index***

*The influence function and the recentered influence function*

The RIF is derived from the influence function (IF). The IF is a specific form of a directional derivative. A directional derivative is used to find the influence of a perturbation or contamination in a distribution, for example from $F_{H}$ towards a new distribution, on a statistic. The IF is the particular form of a directional derivative where the new distribution, denoted as $\delta_{h}$, equals a cumulative distribution function for a probability measure that puts mass 1 at a particular value $h$:

$\delta_{h}(l)$*=*$\left\{ \begin{aligned} 0, &if l<h \\ 1, &if l\geq h \end{aligned} \right.$ , (13)

where $l$ is a draw from $H$. To define the IF of the functional $v(F_{H})$ evaluated at point $h$, denoted as *IF*$(h;v)$, $G_{h}$ is defined as a mixing probability distribution of $F_{H}$and $\delta_{h}$:

$G_{h}=\left( 1-\varepsilon\right)F_{H}+\varepsilon\delta_{h}$*,*  (14)

where $\varepsilon\in(0,1)$ is a probability, or a weight, representing the relative change in the population through the addition of $\delta_{h}.$That is, $G_{h}$ is a distribution that is$\varepsilon$ away from $F_{H}$ in the direction of $\delta_{h}$. IF$(h; v)$ is then defined as:

$IF \left( h; v \right)=\frac{\partial v(G_{h})}{\partial\varepsilon}|_{\varepsilon=0}={lim}_{\varepsilon\to0}\frac{v\left( G_{h} \right)-v(F_{H})}{\varepsilon}$ , (15)

The RIF is a minor transformation of the IF, and is obtained from the IF by adding back the original functional, $v(F_{H})$:

$RIF\left( h;v \right)=v(F_{H})$+$IF \left( h; v \right)$. (16)

*The RIF for a general (bivariate) rank dependent index*

As the rank dependent index, $I$, is a functional of the joint probability distribution $F_{H,F_{Y}}$. In order to get RIF for a bivariate rank dependent index, it is necessary to extend the definitions in Eqs. (13)– (16) from a univariate to a bivariate setting. Let $G_{h, F_{Y}(y)}$ be a bivariate distribution function obtained by an infinitesimal contamination of $F_{H,F_{Y}}$ in both $h$ and $F_{Y}(y)$:

$G_{h, F_{Y}(y)}=\left( 1-\varepsilon\right)F_{H,F_{Y}}+ \varepsilon\delta_{h, F_{Y}(y)}$. (17)

Here $\delta_{h, F_{Y}(y)}$ denotes a joint cumulative distribution function for a joint probability measure that gives mass 1 to$(h, F_{Y}(y))$jointly:

$\delta_{h, F_{Y}\left( y \right)}(l,r)$*=*$\left\{ \begin{aligned} 0, &if l<h or r< F_{Y}(y) \\ 1, &if l\geq h or r> F_{Y}(y) \end{aligned} \right.$ , (18)

where $l$ and $r$ are draws from $H$ and $F_{Y}$ respectively. In analogy with Eq. (15), the bivariate IF of $v^{I}\left( F_{H,F_{Y}} \right)$ evaluated at point (*h,*$F_{Y}(y)$) then is defined as:

$$IF \left( h,F_{Y}\left( y \right);v^{I} \right)=\frac{\partial v^{I}(G_{h, F_{Y}(y)})}{\partial\varepsilon}|_{\varepsilon=0}={lim}_{\varepsilon\to0}\frac{v^{I}\left( G_{h, F_{Y}(y)} \right)-v^{I}(F_{H,F_{Y}})}{\varepsilon} , (19)$$

Finally, the RIF of $I$ is then defined as:

$RIF\left( h,F_{Y}\left( y \right);v^{I} \right)=v^{I}(F_{H,F_{Y}})$+$IF \left( h,F_{Y}\left( y \right);v^{I} \right)$. (20)

The RIF formulas may appear complex; however, they are just a linearization of the statistic. Practical implementation of RIF estimations is straight forward.

***RIF regression decomposition***

Two parameters of interest are estimated using RIF regression: the marginal effect of covariates *X* on a functional, which is an individual effect, and the unconditional partial effect, which is a population effect measure (Heckley et al., 2016). The estimates for these two parameters are as follows.

The recentering of the IF yielding the RIF implies that $v^{I}(F_{H,F_{Y}})$ could be expressed as an expected value of the RIF:

$$v^{I}\left( F_{H,F_{Y}} \right)=\int_{-\infty}^{\infty} RIF(h,F_{Y}\left( y \right);v^{I})\cdot dF_{H,F_{Y}}(h,F_{Y}(y))=E[RIF\left( H,F_{Y};v^{I} \right)] (21)$$

In order to link $v^{I}(F_{H,F_{Y}})$ to the covariates $X$, the law of iterated expectations is applied to express $v^{I}(F_{H,F_{Y}})$ as a conditional expectation [5]:

$$v^{I}\left( F_{H,F_{Y}} \right)=\int_{-\infty}^{\infty} RIF(h,F_{Y}\left( y \right);v^{I})\cdot dF_{H,F_{Y}}(h,F_{Y}(y)) =\int_{-\infty}^{\infty} E[RIF(H,F_{Y};v^{I})|X=x]\cdot dF_{X}(x) (22)$$

where $F_{X}$ is the CDF of $X$.^[[1]](#footnote-2)^ Thus, the decomposition of $v^{I}\left( F_{H,F_{Y}} \right)$ comes down to a problem of estimating a conditional expectation, which can be solved by standard regression methods. Then, for a general function of covariates $X$ and an error term $\epsilon$ , denoted as $\lambda(X,\epsilon)$, the conditional expectation of $RIF(h,F_{Y}\left( y \right);v^{I})$ can be expressed as:

$E\left[ RIF\left( H,F_{Y};v^{I} \right) | X=x \right]=\lambda(X,\epsilon)$ (23)

The first parameter of interest, the marginal effect with respect to$X$, is given by the partial derivative of the regression estimates of (7):

$$\frac{dE\left[ RIF\left( H,F_{Y};v^{I} \right) | X=x \right]}{dx}=\frac{d\lambda(X,\epsilon)}{dx} (24)$$

The second parameter of interest is the unconditional $I$ partial effect, denoted as $\gamma(v^{I})$. It is a vector of average partial derivatives expressed as:

$$\gamma\left( v^{I} \right)=\int_{-\infty}^{\infty} \frac{dE\left[ RIF\left( H,F_{Y};v^{I} \right) | X=x \right]}{dx}\cdot dF_{X}\left( x \right)=\int_{-\infty}^{\infty} \frac{d\lambda(X,\epsilon)}{dx}\cdot dF_{X}\left( x \right) (25)$$

Assuming $\lambda(.)$ to be linear and applying ordinary least square (OLS) to estimate the parameters, can yield an estimator which Heckley et al. (2016) [1] refer to as RIF-CI-OLS. Following Heckley et al. (2016) [1], we used RIF-I-OLS which is both simple and attractive from an operational perspective to conduct decomposition.

RIF-I-OLS identifies our parameters of interest, the marginal effect and the unconditional $I$ partial effect, under the assumptions of additive linearity and zero conditional mean. So the Eq. (8), Eq. (9), and Eq. (10) can be rewritten as:

$E\left[ RIF\left( H,F_{Y};v^{I} \right) | X=x \right]=X^{'}\psi+\mu$ (26)

$$\frac{dE\left[ RIF\left( H,F_{Y};v^{I} \right) | X=x \right]}{dx}=\frac{d[X^{'}\psi+\mu]}{dx}=\psi(27)$$

$$\gamma\left( v^{I} \right)=\int_{-\infty}^{\infty} \frac{d[X^{'}\psi+\mu]}{dx}\cdot dF\left( x \right)=\psi(28)$$

Thus, under the linearity and zero conditional mean assumptions, the marginal effect and the unconditional partial effect are the same and RIF regression is optimally estimated using OLS.

References

1. Heckley G, Gerdtham U-G, Kjellsson G. A general method for decomposing the causes of socioeconomic inequality in health. J. Health Econ. 2016;48:89–106.

2. Erreygers G, Kessels R. Regression-Based Decompositions of Rank-Dependent Indicators of Socioeconomic Inequality of Health. Health Inequal. Emerald Group Publishing Limited; 2013. p. 227–59.

3. Wagstaff A, van Doorslaer E, Watanabe N. On decomposing the causes of health sector inequalities with an application to malnutrition inequalities in Vietnam. J. Econom. 2003;112:207–23.

4. Kjellsson G, Ulf-G G, Dennis P. Lies, damned lies, and health inequality measurements: understanding the value judgments. Epidemiology. 2015;26:673–80.

5. Firpo S, Fortin NM, Lemieux T. Unconditional Quantile Regressions. Econometrica. 2009;77:953–73.

1. Noting that $F_{H,F_{Y}}\left( h,F_{Y}\left( y \right) \right)=\int F_{(H,F_{Y})|X}(h,F_{Y}(y)|X=x)\cdot dF_{X}(x)$, which is substituted into Eq. (22). [↑](#footnote-ref-2)
